# Selective Optical Control of Synaptic Transmission in the Subcortical Visual Pathway by Activation of Viral Vector-Expressed Halorhodopsin

**DOI:** 10.1371/journal.pone.0018452

**Published:** 2011-04-05

**Authors:** Katsuyuki Kaneda, Hironori Kasahara, Ryosuke Matsui, Tomoko Katoh, Hiroaki Mizukami, Keiya Ozawa, Dai Watanabe, Tadashi Isa

**Affiliations:** 1 Department of Developmental Physiology, National Institute for Physiological Sciences, Okazaki, Japan; 2 The Graduate University for Advanced Studies, Hayama, Japan; 3 Department of Biological Sciences, Faculty of Medicine, Kyoto University, Kyoto, Japan; 4 Division of Genetic Therapeutics, Center for Molecular Medicine, Jichi Medical University, Tochigi, Japan; 5 Department of Molecular and Systems Biology, Graduate School of Biostudies, Kyoto University, Kyoto, Japan; University of Oldenburg, Germany

## Abstract

The superficial layer of the superior colliculus (sSC) receives visual inputs via two different pathways: from the retina and the primary visual cortex. However, the functional significance of each input for the operation of the sSC circuit remains to be identified. As a first step toward understanding the functional role of each of these inputs, we developed an optogenetic method to specifically suppress the synaptic transmission in the retino-tectal pathway. We introduced enhanced halorhodopsin (eNpHR), a yellow light-sensitive, membrane-targeting chloride pump, into mouse retinal ganglion cells (RGCs) by intravitreously injecting an adeno-associated virus serotype-2 vector carrying the CMV-eNpHR-EYFP construct. Several weeks after the injection, whole-cell recordings made from sSC neurons in slice preparations revealed that yellow laser illumination of the eNpHR-expressing retino-tectal axons, putatively synapsing onto the recorded cells, effectively inhibited EPSCs evoked by electrical stimulation of the optic nerve layer. We also showed that sSC spike activities elicited by visual stimulation were significantly reduced by laser illumination of the sSC in anesthetized mice. These results indicate that photo-activation of eNpHR expressed in RGC axons enables selective blockade of retino-tectal synaptic transmission. The method established here can most likely be applied to a variety of brain regions for studying the function of individual inputs to these regions.

## Introduction

Multiple inputs regulate neuronal and circuit activities in almost all brain systems. In the early visual system, the superficial layer of the superior colliculus (sSC) receives visual information directly from the retina and indirectly from the primary visual cortex (V1) that receives inputs from the lateral geniculate nucleus (LGN) [for review, see 1]. Electrical stimulation experiments revealed that both inputs drive the activity of sSC neurons [2]–[7]; however, the physiological function of the individual inputs remains unclear. To address this issue, it is necessary to selectively inactivate each input. One way to accomplish this is by lesioning. By lesioning V1, it is possible to inactivate the cortico-tectal input, thus isolating the retino-tectal input. However, such a manipulation may result in plastic changes in the remaining system. For example, V1 lesion studies showed that the retino-tectal connection becomes important for orienting behavior after the lesion, suggesting increased synaptic efficacy ([8], Kato, R. and Isa, T., unpublished observation). On the other hand, it is theoretically impossible to isolate the cortico-tectal input by making a retinal lesion or by pharmacologically blocking the retinal activity because such manipulations disrupt both the retino-tectal and retino-LGN-V1-sSC pathways. Therefore, to selectively suppress the retino-tectal pathway we considered whether a recently developed optogenetic approach could possibly resolve such problems [9], [10].

Halorhodopsin (NpHR) is a yellow light-sensitive, microbial chloride pump derived from *Natronomonas pharaonis* [11]. When expressed in neurons, somatodendritic photo-activation hyperpolarizes the membrane potential and suppresses spiking activities within several milliseconds, leading to reversible changes in animal's behavior [12]–[16]. The fact that photo-activation of axonally expressed channelrhodopsin-2 (ChR2), a blue-light sensitive cation channel, can induce spikes in axons and elicit synaptic responses [17]–[20] prompted us to hypothesize that if axonal NpHR is functional, then it would be possible to inhibit synaptic transmission in a pathway-selective manner with a resolution of milliseconds by expressing NpHR in a target pathway without plastic changes. However, it is still unknown whether photo-activation of NpHR expressed in axons blocks synaptic transmission, and thus, it is important to address this issue. Here we report that photo-activation of the membrane surface-targeting enhanced NpHR (eNpHR) [21], [22], expressed in axons of retinal ganglion cells (RGCs) using an adeno-associated virus serotype-2 (AAV2) vector, selectively inhibits retino-tectal synaptic transmission, resulting in reduction of spiking activities in sSC neurons elicited by visual stimulation in mice. The method established here would be applicable to other brain regions in many species including those in which development of transgenic animals is technically difficult at present.

## Materials and Methods

This study was approved by the Animal Research Committee of Okazaki National Research Institutes (09A206) and Kyoto University (MedKyo∶10044). All efforts were made to minimize the suffering and number of animals used in this study.

### AAV vector preparations

The viral expression construct pAAV-cytomegalovirus promoter (CMV)-eNpHR-enhanced yellow fluorescent protein (EYFP)-woodchuck post-transcriptional regulatory element (WPRE) was made by subcloning the eNpHR-EYFP-WPRE fragment (kindly provided by Dr. K. Deisseroth at Stanford University) into an AAV2 expression cassette, pAAV-MCS (Stratagene). The recombinant vector was co-transfected with a helper plasmid encoding the AAV2 rep/cap genes and an adenoviral helper plasmid into HEK293 cells (#240073, Stratagene) by calcium phosphate co-precipitation. The crude viral lysate was purified by CsCl centrifugation. The final viral concentration was 1.0–1.1×10^13^ genome copies/ml.

### Intravitreous injection of AAV2 vector

C57BL/6 mice, aged 12–13 days and 8–9 weeks, were used for the present *in vitro* and *in vivo* studies, respectively. The mice were anesthetized with a mixture of ketamine (60 mg/kg, ip) and xylazine (10 mg/kg, ip). Under a stereomicroscope, mice received unilateral pressure injections of 1.0–1.5 µl of AAV vector solution into the vitreous body through the sclera region posterior to the limbus using a glass micropipette with a tip diameter of 20–30 µm. After the injection, the pipette was left in place for an additional 30 s and then slowly withdrawn.

### Slice preparation and whole-cell recordings *in vitro*

Three weeks after the injection, the mice were deeply anaesthetized with isoflurane and decapitated. The brains were quickly removed and submerged in ice-cold modified Ringer's solution containing (in mM): 234 sucrose, 2.5 KCl, 1.25 NaH_2_PO_4_, 10 MgSO_4_, 0.5 CaCl_2_, 26 NaHCO_3_, and 11 glucose, and bubbled with 95% O_2_–5% CO_2_ (pH 7.4). Parasagittal slices (300 µm thick) of the SC were cut with a Microslicer (VT1200S, Leica) and incubated at room temperature for >1 h before recording in standard Ringer's solution containing (in mM) 125 NaCl, 2.5 KCl, 2 CaCl_2_, 1 MgCl_2_, 26 NaHCO_3_, 1.25 NaH_2_PO_4_, and 25 glucose, and bubbled with 95% O_2_–5% CO_2_ (pH 7.4). The AAV2-infected retinas were removed, stored in a fixative containing 4% paraformaldehyde in 0.1 M phosphate buffer (PB) (pH 7.2), and processed for histological examination as described below.

Slices were mounted in a recording chamber on an upright microscope (BX-51WI, Olympus) and continuously superfused with the standard Ringer's solution at a flow rate of 2–2.5 ml/min. Whole-cell patch-clamp or cell-attached recordings were obtained from sSC neurons by visual control of patch pipettes, which were prepared from borosilicate glass capillaries and were filled with an internal solution containing (in mM); 150 Cs-gluconate, 5 CsCl, 2 MgCl_2_, 4 Na_2_ATP, 0.3 Na_3_GTP, 10 EGTA, 10 HEPES, 2-4 QX-314, and 0.1 spermine (pH was adjusted to 7.3 with CsOH). To stain the recorded neurons, biocytin (2–4 mg/ml; Sigma) was dissolved in the solution. The resistance of the electrodes was 4–8 MΩ in the Ringer's solutions. The actual membrane potentials were corrected by the liquid junction potential of −10 mV. In voltage clamp recordings we held membrane potentials at −70 mV, aiming to isolate EPSCs. All recordings were performed at 33–34°C. Electrical stimulation was applied as a cathodal square-wave pulse of 200 µs duration with an intensity of up to 100 µA using a glass electrode filled with normal Ringer's solution. Because we often could not firmly trace EYFP-positive RGC fibers that were traveling from the rostral pole of the SC to the recorded cells, we delivered the electrical stimulation to the optic nerve layer of the SC just rostroventral (∼250 µm) to the recorded cells where many eNpHR-positive fibers that would make synaptic contacts with the recorded cells were observed, instead of stimulating the rostral pole of the SC.

To apply yellow laser light to the eNpHR-expressing axons in the sSC, an optical fiber (diameter, 1 mm) was coupled to a 561 nm laser diode (CL561-050-O, CrystaLaser). The fiber output measured under this condition was 36 mW. The fiber was held by a micromanipulator (Narishige) and the tip of the fiber was located ∼500 µm away from the slice surface. By this positioning, not only the eNpHR-positive axons but also recorded cells were illuminated by the laser.

Data were acquired with a Multiclamp 700B amplifier and pClamp10 software (Molecular Devices).

### Visualization of recorded neurons

After recording, the slices were fixed with 4% paraformaldehyde in 0.1 M PB (pH 7.4) for more than a day at 4°C. After fixation, biocytin-filled neurons were visualized by the ABC method (Vectastain, Vector Laboratories). Details are described elsewhere [23], [24].

### Visual stimulation and single-unit recordings *in vivo*

Four to five weeks after the vector injection, mice were anesthetized with urethane (1.2–1.5 g/kg in saline, i.p.) and their heads were placed in a stereotaxic apparatus (Narishige). Dexamethasone (2.0 mg/kg) was injected subcutaneously. Additional urethane (0.2–0.3 g/kg) was administered as needed. The incision was infiltrated with lidocaine (xylocaine jelly). The animal's body temperature was maintained at 37°C by a rectal thermoprobe feeding back to a heating pad (BWT-100, Bioresearch Center). Heart rate was monitored continuously throughout the experiment. The eye was covered with silicone oil. After performing a craniotomy to expose the cortex overlying the SC, the exposed area was covered with agarose (2% in saline). A glass electrode (10–15 MΩ, filled with 0.5 M KCl) was vertically lowered into the brain using a micromanipulator, until visual responses to nearly whole visual field stimuli were detected. After entering the sSC, the glass electrode was slowly advanced and single unit activities were recorded. Electrical signals were bandpass-filtered (0.3–6 kHz) and amplified with a Multiclamp 700B amplifier (1,000X, Molecular Devices) before being captured on a computer using an analog-to-digital card (National Instruments).

Visual stimuli were generated by Matlab (Mathworks) programs using the Psychophysics Toolbox extensions [25], [26]. The stimuli were displayed on a 17-inch LCD monitor placed 25 cm from the mouse's eye contralateral to the recorded hemisphere. The eye ipsilateral to the recorded hemisphere was covered. To determine the receptive field of sSC neurons, 6° light circles (55–60 cd/m^2^) were flashed at different locations on a gray background (6–8 cd/m^2^), and then spike rate during the 1 s response to both flash onset and offset were calculated for each location. A custom-made program using MatLab (Mathworks) generated a peristimulus time histogram (PSTH) from all the responses to visual stimulation. The center of the receptive field was defined as the location where the maximum number of spikes per second was observed in the PSTH. After determining the center of the receptive field, light circles (6–12° in diameter) were presented for 300 ms. An inter-stimulus interval (duration, 5 s) was introduced to minimize cell adaptation. The number of spikes was counted to calculate the response to each stimulus. Spikes occurring in the first 30–330 ms after turning on the stimulus were binned as part of the ON response of the cell, and subsequent spikes (330–1230 ms) were binned into the OFF response.

To apply yellow laser light to the sSC, an optical fiber (diameter, 500 µm) was coupled to a 561 nm laser diode (CL561-050-O, CrystaLaser). The fiber output measured under this condition was 20 mW. The fiber held by a micromanipulator, which was different from the manipulator that held the recording electrode, was lowered obliquely into the cortex (∼0.5 mm in depth) overlying the sSC ∼0.5 mm rostral and ∼0.15 mm lateral to the recording site. The angle of the fiber was adjusted so that the laser illumination covered the recording site.

The mice were killed at the end of recordings with an overdose of sodium pentobarbital (80 mg/kg, i.p.), perfused transcardially with 0.05 M phosphate buffered saline (PBS, pH 7.4) followed by fixative containing 4% paraformaldehyde in 0.1 M PB (pH 7.4). The brains and eyes were removed, postfixed for 1–2 days, and processed for histological examination as described below.

### Data analysis

Data obtained *in vitro* and *in vivo* were analyzed with Clampfit (Molecular Devices) and Matlab (Mathworks), respectively. All values are expressed as mean ± SEM. Statistically significant differences were examined with a two-tailed Student's paired *t*-test and were considered significant if *P*<0.05.

### Histology

To confirm the expression of eNpHR-EYFP-positive axons in the sSC, the brains removed after *in vivo* recordings were cryoprotected in 30% sucrose in 0.1 M PB (pH 7.4), and 50–100-µm-thick coronal or sagittal sections were cut on a freezing microtome. The expression of eNpHR-EYFP in RGCs was examined in whole-mount retinas and retinal vertical sections (16-µm thickness). Fluorescence was observed and photographed with digital microscopes (Axioplan2, Zeiss and BIOREVO, BZ-9000, Keyence) or with a confocal microscope system (Fluoview 300, Olympus).

To calculate the percentage of eNpHR-expressing RGCs, the retinal vertical sections were immunohistochemically stained by a neuronal cell maker NeuN. The sections were blocked in PBS with 10% normal goat serum (NGS) and 0.1% Triton X-100 at room temperature and reacted with mouse monoclonal anti-NeuN antibody (1∶400; Millipore) in PBS with 2% NGS and 0.1% Triton X-100 at 4°C overnight. The sections were then washed four times in PBS with 0.1% Triton X-100 and incubated with Alexa Fluor 594-conjugated anti-mouse IgG (1∶200; Invitrogen) in PBS with 2% NGS and 0.1% Triton X-100 at room temperature for 3 h. After washing four times in PBS, the sections were coverslipped with SlowFade Antifade Kit (Invitrogen). Fluorescence was observed and photographed with digital microscopes (Axioplan2, Zeiss). For quantification of the percentage of eNpHR-expressing RGCs, we chose five sections from each of three retinas, in which eNpHR-expressing RGCs were relatively densely observed, and counted the number of NeuN-positive cells as RGC number and also counted the number of EYFP-positive cells as eNpHR-expressing cell number in the whole area of each section.

## Results

### Expression of eNpHR in the retina and sSC

**Figure 1:**
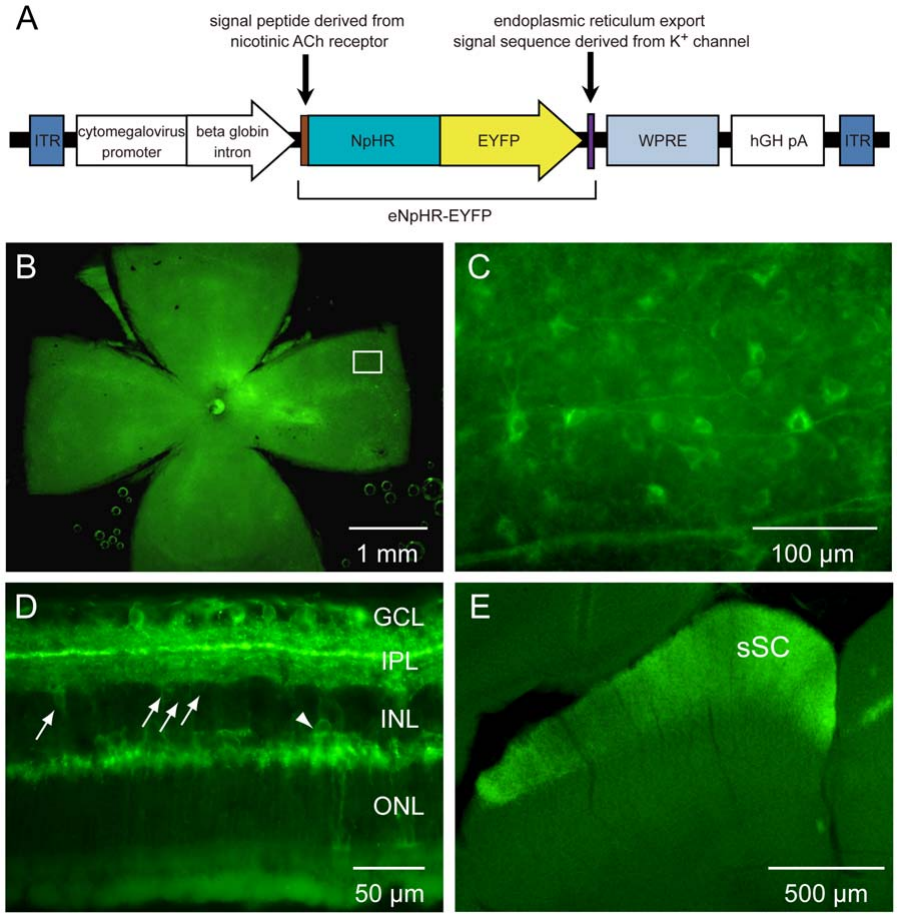
Expression of eNpHR-EYFP in retina and sSC. *A*, The AAV-CMV-eNpHR-EYFP-WPRE expression cassette: ITR: inverted terminal repeat; CMV: cytomegalovirus promoter; eNpHR: enhanced halorhodopsin; EYFP: enhanced yellow florescent protein; WPRE: woodchuck post-transcriptional regulatory element; hGH pA: human growth hormone polyadenylation sequence. *B,C*, eNpHR-EYFP fluorescence images taken from retinal whole-mount at low (*B*) and high (*C*) magnification. The rectangular area in *B* is enlarged in *C*. *D*, eNpHR-EYFP fluorescence image taken from retinal vertical section. EYFP-positive ganglion cells and axons were observed in the GCL. Arrows point to infected amacrine cells and an arrowhead points to an infected horizontal cell. *E*, eNpHR-EYFP fluorescence image taken from the superficial layer of the superior colliculus (sSC) in coronal section. Dense EYFP-positive RGC-derived axons were observed throughout the medio-lateral axis. GCL: ganglion cell layer; INL: inner nuclear layer; IPL: inner plexiform layer; ONL: outer nuclear layer.

To introduce eNpHR into RGCs, we used the AAV2 vector with a CMV (Fig. 1*A*), based both on our preliminary data showing that AAV2 is the most effective expression vector among AAV1, 2 and 5, which we tested (data not shown), and on previous reports demonstrating that AAV2 as the preferable carrier vector for RGCs in rodents [27–29, but see 30]. We first histologically confirmed the expression of eNpHR in RGCs via the fluorescence of EYFP. As shown in Figure 1*B*, three to four weeks after the intravitreous injection, bright green fluorescence was observed in the whole-mounted retina with intermittently dense and scarce fluorescence. At high magnification, many EYFP-positive RGCs and axons were observed at the focus level of RGC layers (Fig. 1*C*). In retinal vertical sections bright fluorescence was also confirmed in ganglion cells and in the inner plexiform layer (IPL), suggesting both ON and OFF ganglion cells express eNpHR (Fig. 1*D*). We frequently observed EYFP signals in amacrine cells (Fig. 1*D*, arrows) and occasionally in horizontal cells (Fig. 1*D*, arrowhead) and bipolar cells (data not shown) as reported previously in AAV-ChR2 and AAV-HaloR (another abbreviation of NpHR) injection studies [28], [29].

To quantify the percentage of eNpHR-expressing RGCs, we immunohistochemically stained retinal vertical sections with a neuronal cell maker NeuN (Fig. S1) and selected the sections in which relatively dense EYFP fluorescence was observed. We then counted the number of NeuN-positive and EYFP-eNpHR-positive cells in the RGC layer of five sections obtained from each of three retinas (a total of 15 sections). We found a very high expression (>80%) of eNpHR in many retinal regions, a moderate expression (60 ∼ 80%) in some areas, and a slightly lower expression (∼ 60%) in a few regions (Table 1).

**Table 1:** Percentage of eNpHR-expressing retinal ganglion cells.

	Retina A			Retina B			Retina C		
Section #	NeuN	eNpHR	%	NeuN	eNpHR	%	NeuN	eNpHR	%
1	318	220	69.2	344	298	86.6	314	221	70.4
2	354	289	81.6	320	308	96.3	352	193	54.8
3	349	282	80.8	354	298	84.2	297	223	75.1
4	308	268	87.0	333	303	91.0	319	216	67.7
5	314	229	72.9	301	265	88.0	302	224	74.2
Total	1643	1288	78.4	1652	1472	89.1	1584	1077	68.0

Consistent with the findings in the retina, we also confirmed the presence of eNpHR-EYFP-expressing RGC axon terminals in the sSC. As revealed by the EYFP fluorescence, dense axonal expression of eNpHR was observed throughout the sSC layers, although intermittently dense and scarce expression regions were detected, suggesting that the expression level was not homogenous over the retina (Fig. 1*E*).

### Laser illumination blocks retino-tectal synaptic transmission

**Figure 2:**
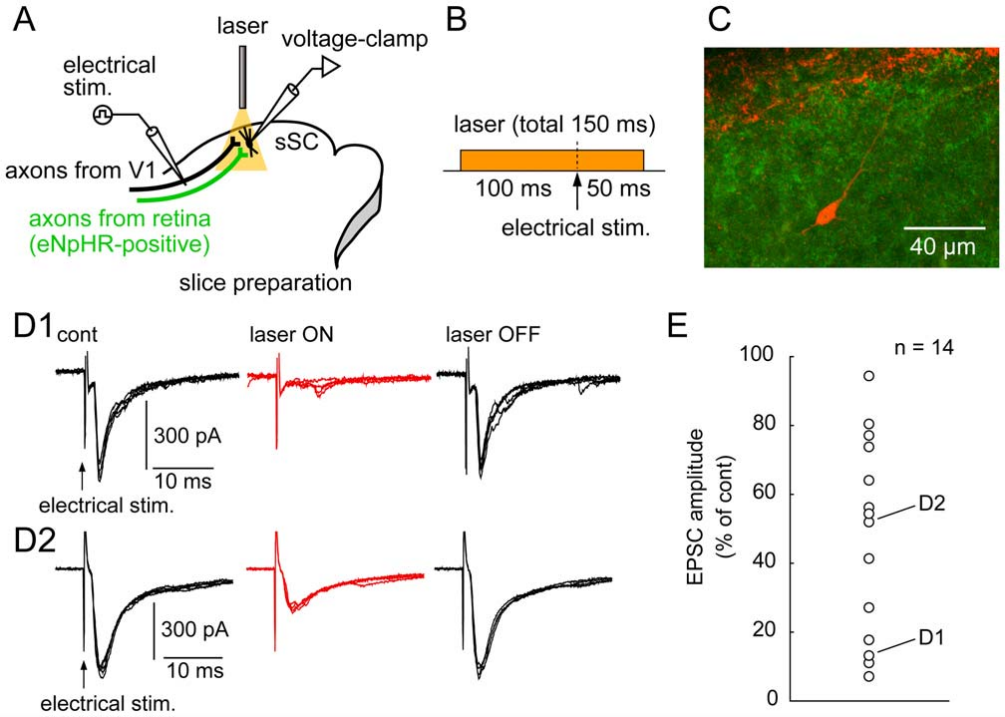
Photo-activation of axonally expressed eNpHR suppresses retino-tectal synaptic transmission. *A*, Schematic of experimental configuration. Electrical stimulation was applied to the optic nerve layer, which included both retino-tectal and cortico-tectal axons. Whole-cell recordings were obtained from sSC neurons. Yellow laser illuminated the eNpHR-positive axons and the recorded cells. *B*, Timing and duration of laser illumination. *C*, A representative photograph of a recorded sSC cell (red) surrounded by EYFP-positive axons (green). *D*, Examples of laser illumination-induced suppression of EPSCs. EPSCs evoked by electrical stimulation in the absence (cont, black), presence (laser ON, red), and again in the absence (laser OFF, black) of laser illumination, in cells showing nearly complete (*D*1) and partial (*D*2) suppression. In each condition 5 traces were superimposed. *E*, Summary of suppression of EPSCs in 14 sSC neurons.

Next, we examined whether photo-activation of axonally expressed eNpHR blocks retino-tectal synaptic transmission at the axon terminals in the sSC. Parasagittal slices including the sSC were obtained from mice that received an intravitreous injection of AAV2 vector three weeks before the experiments. We performed whole-cell recordings from sSC neurons that were surrounded by dense eNpHR-positive axons (Fig. 2*A,C*). Electrical stimulation was applied to the optic nerve layer, where the RGC axons are located, and eNpHR was activated by illuminating fluorescence-positive axons with an optical fiber coupled to an yellow laser diode (see Materials and Methods). Figure 2*D* *1* illustrates one example of the effect of laser illumination on whole-cell recorded currents. Electrical stimulation evoked EPSCs in the absence of laser illumination (Fig. 2*D* *1*, *left*). Yellow laser illumination applied to the slice (probably illuminating both the recorded neuron and surrounding axons) from 100 ms before to 50 ms after the electrical stimulation (Fig. 2*B*), clearly reduced the amplitude of the evoked EPSCs (Fig. 2*D* *1*, *middle*). The suppression disappeared immediately after turning off the laser (Fig. 2*D* *1*, *right*). In another example, laser illumination partially suppressed the EPSCs (Fig. 2*D* *2*). The degree of suppression varied from cell to cell (Fig. 2*E*), presumably depending on various factors discussed later. In any case, these results indicate that photo-activation of eNpHR expressed in axons reversibly inhibits synaptic transmission. To investigate properties of the laser illumination-induced suppression in more detail, we further analyzed cells that exhibited >40% inhibition in the next series of experiments.

**Figure 3:**
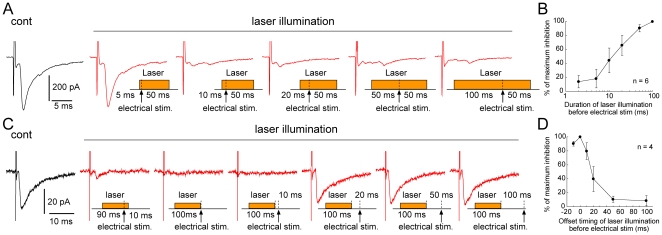
Blockade of synaptic transmission depends on duration and timing of laser illumination. *A*, Example traces of the photo-inhibition of EPSCs with different durations of laser illumination before electrical stimulation. Control (black) stimulation without laser illumination and following various durations of laser illumination beginning from 5–100 ms before electrical stimulation and lasting until 50 ms after stimulation (red). *B*, Population data for six cells showing the effectiveness of laser illumination durations before electrical stimulation for blocking EPSCs. *C*, Example traces of photo-inhibition of EPSCs with different timings of laser illumination relative to electrical stimulation. Control (black) stimulation in the absence of laser illumination and traces following 100-ms laser illuminations delivered beginning 90 ms, 100 ms, 110 ms, 120 ms, 150 ms, and 200 ms before electrical stimulation (red). *D*. Summary data for four cells showing the effectiveness of different timings of electrical stimulation, relative to the offset of laser illumination, for blocking synaptic transmission.

We next examined the most effective duration and timing of laser illumination, relative to electrical stimulation, for suppressing synaptic transmission. To determine the most effective duration of the laser illumination we altered its duration before electrical stimulation and maintained a constant post-stimulus illumination (50 ms). Laser illumination that started 5 ms before electrical stimulation did not affect the evoked EPSCs (Fig. 3*A*, *second trace from the left*). However, in some neurons (3 of 6 cells), laser illumination that started just 10 ms before electrical stimulation was effective enough to suppress synaptic transmission (Fig. 3A, *third trace from the left*). Laser illumination that started more than 20 ms prior to the electrical stimulation was also effective in almost all of the neurons (Fig. 3A, *right three traces*). Figure 3*B* depicts the population data indicating that laser illumination applied >20 ms before the electrical stimulation may be necessary to block the synaptic transmission effectively.

To address the effective timing of laser illumination, we maintained a constant duration (100 ms), but varied the time, relative to the electrical stimulation, for delivering the illumination. As shown in Figure 3C, laser illumination applied from 90 ms before to 10 ms after the electrical stimulation effectively blocked EPSCs (Fig 3*C*, *second trace from the left*). Illumination applied immediately prior to the electrical stimulation was also effective (Fig 3*C*, *third trace from the left*). Interestingly, 100-ms illumination delivered 10 ms prior to the electrical stimulation still markedly suppressed EPSCs (Fig 3*C*, *fourth trace from the left*). However, laser illumination that ended more than 20 ms before the electrical stimulation no longer affected EPSCs (Fig. 3*C*, *right three traces*). Taken together, these data indicate that 100-ms illumination ending at maximum 10 ms before the electrical stimulation ensures the suppression of synaptic transmission under our *in vitro* experimental conditions.

**Figure 4:**
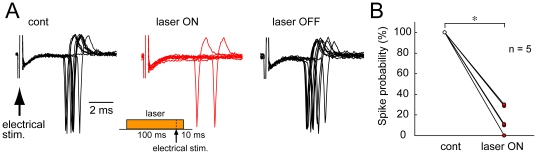
Photo-activation of axonal eNpHR inhibits spike activity in sSC neurons *in vitro*. *A*, Left, In control condition, every optic nerve stimulation reliably evoked spikes recoded by cell-attached configuration in slice preparation. Middle, Laser illumination applied from 100 ms before to 10 ms after electrical stimulation (inset) suppressed the spike activity. Right, The reduced spike activity was recovered by turning off the laser. In each condition 10 traces were superimposed. *B*, Summary plot of spike probabilities following laser illumination for five cells. *P<0.001.

In addition to the effects of laser illumination on the evoked currents in sSC neurons, we also investigated its effects on the spiking activity of sSC neurons *in vitro* by performing cell-attached recordings. We applied electrical stimulation to the optic nerve layer and chose a stimulus intensity that elicited spikes with a probability of 100% (Fig. 4*A*, *left*). Laser illumination effectively suppressed the spiking activity, reducing the spike probability to 15.9±5.7% (*P*<0.001, n = 5) (Fig. 4*A*, *middle*, and *B*). The reduced activity recovered immediately after turning off the laser (Fig. 4*A*, *right*). These results indicate that eNpHR-mediated suppression of retino-tectal synaptic transmission is powerful enough to inhibit the spiking activity of sSC neurons.

### Laser illumination reduces visual stimulation-induced activity in sSC neurons *in vivo*

**Figure 5:**
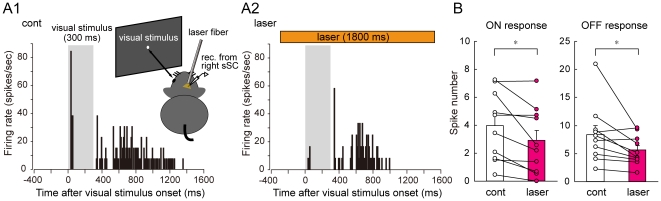
Laser illumination partially, but significantly blocked sSC-spiking activities induced by visual stimulation *in vivo*. *A*, PSTHs showing spiking activity evoked in a cell by visual stimulation (300 ms duration) during control (cont; *A1*) and laser illumination onto the sSC surface before, during, and after the visual stimulation (from −300 to 1500 ms after the onset of visual stimulation) (laser; *A2*). Hatched areas represent the duration of visual stimuli. Inset: Schematic of experimental setup for extracellular recordings and laser illumination *in vivo*. *B*, Population data from 10 cells depicting the effects of laser illumination on both ON and OFF responses to visual stimuli. *P<0.05.

We finally examined whether the suppression of retino-tectal synaptic transmission with photo-activation of eNpHR is applicable to the *in vivo* sSC. For this purpose, we made extracellular, single-unit recordings from sSC neurons in anesthetized mice that had received injections of the vector more than four weeks before the experiments (n = 3). After determining the receptive field of the recorded cells by flashing visual stimuli on a display placed in front of the mice, we applied appropriately sized visual stimuli (6–12°, 300 ms) in the center of the receptive field. Figure 5*A* shows examples of PSTHs in a cell, in which clear ON and OFF responses were elicited by visual stimulation (Fig. 5*A*1). To illuminate a large area of the sSC, an optical fiber (500 µm in diameter) was inserted into the cortex that overlaid the sSC. Laser illumination then applied from 300 ms before to 1500 ms after the onset of visual stimulation markedly reduced both the ON and OFF responses (Fig. 5*A*2). Figure 5*B* shows the population data recorded from 10 sSC neurons. Laser illumination significantly reduced the number of spikes evoked in ON timing (30–330 ms after the onset of visual stimulus; 3.9±0.7 in controls, 2.8±0.7 in laser illuminated, *P*<0.05) and OFF timing (330–1230 ms after the onset of visual stimulus; 8.3±1.5 in controls, 5.5±0.7 in laser illuminated, *P*<0.05). Thus, these results demonstrate that photo-activation of eNpHR expressed in RGC axons synapsing onto sSC neurons suppresses visual stimulus-induced spiking activity of sSC neurons *in vivo*.

## Discussion

We have developed a novel method to selectively suppress retino-tectal synaptic transmission, both *in vitro* and *in vivo*, by photo-activating eNpHR expressed in RGC axons by means of a viral vector. This is the first evidence indicating that photo-activation of axonal NpHR inhibit synaptic transmission, which would have been enabled by employing the membrane-targeting eNpHR generated by Gradinaru et al. [21].

### Methodological considerations

Our method is superior to other commonly used perturbation techniques, such as lesioning and pharmacologically blocking particular inputs in several points. First, in lesion experiments, plastic changes in synaptic or circuit level are inevitable. Indeed, V1 lesion studies have suggested an enhanced functional importance of the sSC in orienting behavior ([8], Kato, R. and Isa, T., unpublished observation), reflecting compensatory mechanisms. Thus, interpreting the effects of a given lesion is difficult because the possibilities that the effect reflects the absence of the lesioned input or the consequence of compensatory mechanisms cannot be clearly distinguished from one another. Second, pharmacological blockade is limited in spatio-temporal aspects. The control of drug spread that determines the blocked region is quite difficult and temporally precise manipulation is almost impossible. In addition, because drug effect usually lasts at least several tens of minutes, the possibility cannot be ruled out that plastic changes may occur during that time period. Finally, because lesion or pharmacological blockade of the retina abolishes all visual information processing via both the retino-tectal and retino-LGN-V1-sSC pathways, these manipulations cannot be applied to the retina to study the physiological functions of each pathway. In sharp contrast, our optogenetic method overcomes these problems. As the duration of laser illumination is at most several seconds, plastic changes are unlikely to occur. Moreover, because our strategy is to express eNpHR in specific axons by means of a viral vector, pathway-selective inhibition is possible without affecting either other inputs or retinal function.

In this study we employed a commonly used promoter, CMV, to drive the expression of eNpHR in RGCs. Because of its non-specific profile [28], [29], not only RGCs but also other cell types expressed eNpHR following intravitreous injection of the viral vectors (Fig. 1*D*). However, as we aimed to express opsins in axons and to photo-activate them in terminal regions, we did not need to consider the expression of eNpHR in other retinal cell types that do not innervate the sSC.

### Synaptic inhibition *in vitro*

We occasionally found that the laser illumination almost completely blocked the evoked EPSCs (Fig. 2*D*1). This clearly demonstrates that if only eNpHR-positive axons were stimulated, synaptic transmission mediated by the axons would be almost completely blocked by the laser illumination. On the other hand, we often observed partial suppression of the evoked EPSCs. This may be accounted for by a few possibilities. First, because electrical stimulation in the optic nerve layer may activate both eNpHR-expressing retinal axons and V1-derived eNpHR non-expressing axons, the laser illumination-resistant component of the EPSCs may have been caused by V1-derived synaptic input. Second, because not all RGCs expressed eNpHR under our experimental condition, if we stimulated eNpHR-negative RGC axons, laser illumination did not block the synaptic transmission from those axons. With regard to this point, infection efficacy should be improved in future studies. Third, we cannot exclude the possibility that electrical stimulation applied to the optic nerve layer excited axon collaterals of sSC neurons, which did not express eNpHR.

We found that to suppress the synaptic transmission effectively, laser illumination should be started about 20 ms before the electrical stimulation (Fig. 3*A,B*). Also, if the duration of the illumination was long enough, turning off the laser 10 ms before the electrical stimulation still effectively inhibited synaptic transmission (Fig. 3*C,D*). We consider that these time lags between laser illumination and its suppressive effect may reflect the kinetics of axonally expressed eNpHR. Based on previous studies showing that it takes several tens of milliseconds to reach a maximum membrane hyperpolarization after the onset of laser illumination to NpHR-expressing neurons [12], [15], we speculate that it might take about 20 ms from the onset of laser illumination to reach a hyperpolarized, threshold membrane potential at which spike generation or propagation along axons may be suppressed, and that about 10 ms might be necessary to recover from the threshold potential under our experimental condition.

It has been reported that, in sSC neurons, part of the cortical inputs are mediated by NMDA receptors and that retino-tectal inputs that activate non-NMDA receptors and depolarize the membrane potential may contribute to this NMDA receptor-mediated excitation [4], [31]. Thus, when the non-NMDA receptor-mediated inputs are blocked even partially, the resultant impact on spike activity may be large. In support of this hypothesis, the effect of laser illumination on spike activity recorded with the cell-attached configuration seemed to be more remarkable than that on evoked currents recorded with the voltage-clamp.

### Synaptic inhibition *in vivo*

The suppression of visual stimulus-induced spike activities with laser illumination was most likely the consequence of inhibiting retino-tectal synaptic transmission, even though the inhibitory effect was partial. Although we did not yet examine the functional role of the retino-tectal and cortico-tectal inputs under physiological conditions, inhibition of synaptic transmission in the retino-tectal pathway is the most important finding of the present study, as previous studies were unable to accomplish this selective inhibition *in vivo* as discussed above. The remaining spike activities may be ascribable to several possibilities. First, because we did not eliminate the V1 so as not to induce plastic changes, the remaining activities might be mediated by cortical input. In support of this hypothesis, previous studies reported that visual stimulation to anesthetized mice elicited spiking activity in V1 neurons [32]–[35]. Second, the neurons exhibiting illumination-resistant spikes might not receive eNpHR-expressing retinal input. As discussed above, the expression of eNpHR was observed in a population of some, but not all, RGCs. Therefore, if recorded neurons received inputs from eNpHR-negative RGC axons, spike activities of such sSC neurons may not disappear with the laser illumination. Third, it is possible that laser illumination of the surface of the sSC might not be strong enough to activate axonal eNpHR within the sSC. However, this is unlikely because Gradinaru et al. [14] demonstrated that the intensity of their laser, which was weaker than that of our laser, was strong enough to activate opsin-expressing neurons at a depth of over 1 mm in brain tissue. Although we did not measure the power of the laser at the surface of the sSC *in vivo*, the fact that thickness of the sSC in mice is ∼300 µm led us to speculate that the laser could activate eNpHR-positive fibers in the sSC.

Although the degree of laser effects on On and Off responses was not statistically different (On, 62.9±10.9% of control vs Off, 72.9±6.9% of control, *P*>0.05), the CV values of the changes in On response (0.55) and Off response (0.29) were different, implicating larger variability in On response than in Off response. At present, we cannot identify the reason for this difference. One possibility might be ascribable to different expression level of eNpHR in On- and Off-type ganglion cells. On and Off responses in sSC neurons observed in the present study might have been caused by inputs mainly from On- and Off-type RGCs. Thus, if the expression level of eNpHR between these RGC types was different, the effect of laser illumination on the On and Off responses may be discrete. Further studies will be required for clarifying this point.

### Application to other brain regions and to other experimental animals

The method we established here would be useful for analyzing not only retino-tectal vs retino-LGN-V1-sSC pathways, but also other brain regions that receive multiple inputs from different sources. For example, the striatum receives two major afferent inputs: from the cortex and the thalamus [36], [37]. Although these two inputs can be separately activated by different stimulation sites in the slice preparation [38], our method would be advantageous under physiological conditions *in vivo*.

To more fully understand integrative brain functions, it may be necessary to use non-human primates as animal models and to combine molecular engineering techniques. However, at present, the range of transgenic techniques applicable to non-human primates is still limited [39]. Under such conditions, the expression of eNpHR in specific brain regions using viral vectors should be quite useful. For example, if we were to compare visually guided behaviors in non-human primates expressing eNpHR in their retino-tectal and retino-LGN axons following laser illumination in the sSC or LGN, it would be possible to study the role of the geniculo-striate and retino-tectal pathways in these behaviors. Thus, we believe that pathway-selective inhibition, uncontaminated by synaptic plasticity, would be a powerful tool to investigate neuronal circuit mechanisms related to higher brain functions.

## Supporting Information

Figure S1 **Photomicrographs showing eNpHR expression in retinal ganglion cells.** eNpHR-EYFP (*Left*), NeuN-Alxa Fluor 594 (*Middle*) fluorescence, and merged (*Right*) images taken from a vertical section of the retina. Arrowheads indicate double-labeled RGCs.
(TIF)
