# Supplementary figures and images for: Selective Optical Control of Synaptic Transmission in the Subcortical Visual Pathway by Activation of Viral Vector-Expressed Halorhodopsin
Source: PLoS One. 2011 Apr 5;6(4):e18452. doi: 10.1371/journal.pone.0018452 (PMC3071716; doi:10.1371/journal.pone.0018452)

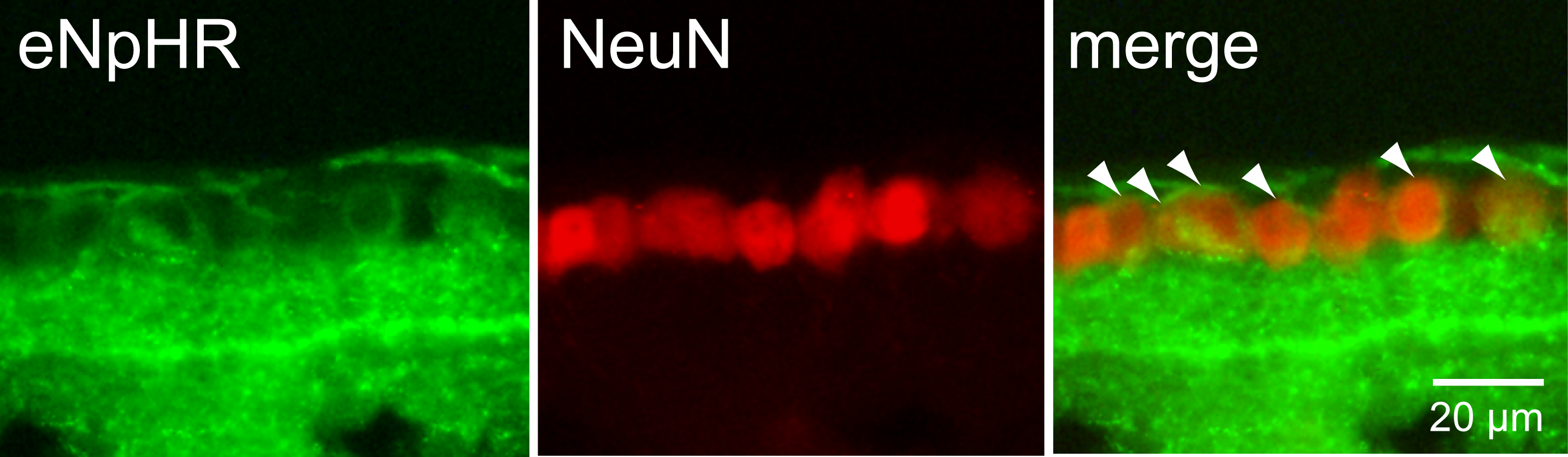

Supplement: Figure S1 — Photomicrographs showing eNpHR expression in retinal ganglion cells. eNpHR-EYFP (Left), NeuN-Alxa Fluor 594 (Middle) fluorescence, and merged (Right) images taken from a vertical section of the retina. Arrowheads indicate double-labeled RGCs. (TIF) [file pone.0018452.s001.tif]
